# Src family kinase activity drives cytomegalovirus reactivation by recruiting MOZ histone acetyltransferase activity to the viral promoter

**DOI:** 10.1074/jbc.RA119.009667

**Published:** 2019-07-04

**Authors:** Liane Dupont, Lily Du, Madeleine Poulter, Stephanie Choi, Megan McIntosh, Matthew B. Reeves

**Affiliations:** Institute of Immunity and Transplantation, Division of Infection and Immunity, Royal Free Hospital, University College London, Hampstead, London NW3 2PF, United Kingdom

**Keywords:** cell signaling, herpesvirus, gene expression, histone acetylation, chromatin modification, cytomegalovirus reactivation, epigenetic regulation, hematopoietic cell kinase, lysine acetyltransferase 6A (KAT6A), phosphoproteomics

## Abstract

Human cytomegalovirus (HCMV) latency and reactivation rely on a complex interplay between cellular differentiation, cell signaling pathways, and viral gene functions. HCMV reactivation in dendritic cells (DCs) is triggered by IL-6 and extracellular signal-regulated kinase (ERK)–mitogen-activated protein kinase signaling. However, activation of the same pathway fails to reactivate HCMV in other myeloid cell types, despite this signaling axis being active in those cells. We hypothesized that IL-6–induced ERK activation initiates the changes in chromatin structure required for viral reactivation but that a concomitant signal is necessary to complete the changes in chromatin structure required for gene expression to occur. Using a differential phosphoproteomics approach in cells that do or do not support IL-6–induced viral reactivation, we identified the concomitant activation of an Src family kinase (SFK), hematopoietic cell kinase (HCK), specifically in DCs in response to IL-6. Pharmacological and genetic inhibition of HCK activity indicated that HCK is required for HCMV reactivation. Furthermore, the HCK/SFK activity was linked to recruitment of the monocytic leukemia zinc finger protein (MOZ) histone acetyltransferase to the viral promoter, which promoted histone acetylation after ERK-mediated histone phosphorylation. Importantly, pharmacological and genetic inhibition of MOZ activity prevented reactivation. These results provide an explanation for the selective activation of viral gene expression in DCs by IL-6, dependent on concomitant SFK and ERK signaling. They also reveal a previously unreported role for SFK activity in the regulation of chromatin structure at promoters in eukaryotic cells via MOZ histone acetyltransferase activity.

## Introduction

The regulation of eukaryotic gene expression requires the coordinated action of a number of molecular mechanisms active within the cell. One key mechanism is selective activation of cellular signaling pathways to either activate or repress gene expression in response to cell stimulation (1). It is noteworthy that a major contribution to the regulation of more than 20,000 human genes depends on a relatively small number of canonical signaling pathways (2). Thus, how such complex patterns of gene regulation are performed by a limited number of pathways is an area of great interest. The key players in these pivotal signaling pathways have mostly been identified and characterized (2). Thus, even more prescient is the question of how a particular pathway can evoke different effects depending on the stimulus and cell type in which it is being activated (1). This alone suggests that the molecular and cellular context in which the pathway is being activated is critical for dictating the final output of the effector function.

We consider this problem through studies of HCMV^3^ reactivation. Lifelong latent HCMV infections are established in CD34+ hematopoietic progenitor cells, where a pivotal event is silencing of lytic viral gene expression, with regulation of the major immediate-early promoter (MIEP) hypothesized to be a critical determinant (3–6). Consequently, a key first step toward reactivation is the induction of major *IE* gene expression from this previously silenced promoter; thus, research has focused on the factors regulating this. This has led to the consensus that cellular differentiation of naturally latent HCMV CD34+ cells or monocytes is pivotal for robust reactivation (5–13). Furthermore, reactivation driven by cellular differentiation is augmented by inflammatory cytokine signaling, supporting key roles for cellular signaling (5, 8, 14, 15). It is likely that this is multi-faceted, with ERK–MAPK, cAMP, and epidermal growth factor receptor/PI3K signaling implicated in HCMV reactivation from latency in a cell- and model-specific manner (8, 16–19). What is consistent is that reactivation is largely dependent on cellular mechanisms of gene regulation through the complimentary mechanisms of mimicry (the MIEP bears the hallmarks of an inflammatory promoter (20)) and direct viral regulation (*e.g.* UL135/UL138 antagonism and epidermal growth factor receptor (17)). Ultimately, the HCMV genome is regulated by higher-order chromatin structure and, essentially, can be considered another eukaryotic genetic unit for the purpose of regulatory studies.

Here we show that ERK–MAPK–driven reactivation of HCMV viral gene expression depends on the concomitant activation of Src family kinase (SFK) signaling in monocyte-derived dendritic cells (MoDCs). We link this to the activation of SFK member hematopoietic cell kinase (HCK), which is highly expressed in cells that support reactivation; consequently, its deletion from cells is sufficient to abrogate reactivation. Through molecular studies, we show for the first time that SFK signaling can drive histone acetylation and does this, in part, via recruitment of the histone acetyltransferase MOZ to the MIEP. Thus, SFK activity works after ERK-MAPK signaling to potentiate an open chromatin structure, ensuring that reactivation proceeds after exit from latency.

## Results

### IL-6 promotes increased phosphorylation of Src family kinases in MoDCs

**Figure 1.**
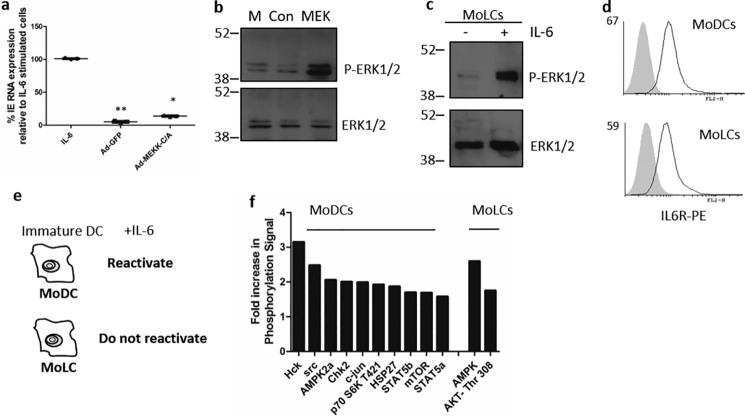
**IL-6 promotes distinct signaling signatures in DC subtypes.** *a*, qRT-PCR to measure IE gene expression in MoDCs 16 h after stimulation with IL-6 or 2 days after transduction with adenovirus vectors expressing constitutively active MEK or the GFP control. *b*, Western blot for ERK phosphorylation in MoDCs (*M*) or MoDCs transduced with adenoviruses expressing GFP (*Con*) or constitutively active MEK. *c*, Western blot for total ERK and ERK phosphorylation in MoLCs left unstimulated (−) or stimulated with IL-6 (+). *d*, flow cytometric analysis of MoDCs and MoLCs stained with isotype (*filled*) or IL-6Ra–phycoerythrin (*PE*, *line*) antibodies. *e*, schematic of differential IL-6 responses in DC subtypes. *f*, summary of differentially phosphorylated proteins in IL-6–stimulated MoDCs and MoLCs. *, *p* < 0.05; **, *p* < 0.01.

We have observed previously that IL-6–ERK–MAPK signaling promotes HCMV reactivation in MoDCs but not undifferentiated progenitors (8), and, consistent with this, delivery of a dominant negative version of the ERK1/2 kinase MEK1 (an inhibitor of ERK1/2 activation) inhibits reactivation (16). Thus, our first experiments tested the reciprocal experiment: delivery of a constitutively active version of MEK1. The data showed that activation of ERK by delivery of a constitutively active version of MEK kinase does not trigger the full induction of viral IE gene expression (Fig. 1*a*), despite evidence of increased ERK phosphorylation in the cells (Fig. 1*b*). This, in the context of our previous results, suggested that activation of ERK alone was not sufficient for induction of viral gene expression.

To investigate this further, we took advantage of a previous study we had performed, in which we demonstrated that, when we altered the culture conditions and promoted monocytes to differentiate into immature monocyte-derived Langerhans-like cells (MoLCs), they did not support IL-6–induced reactivation, unlike what was observed in MoDCs (21). This suggested that the pathway of differentiation was playing an important role in the reactivation phenotype. However, an analysis of MoLCs demonstrated that they were clearly IL-6–responsive so that IL-6 promoted ERK phosphorylation in MoLCs (Fig. 1*c*), which was consistent with the observation that both MoDCs and MoLCs express similar levels of the IL-6 receptor on their cell surface (Fig. 1*d*). All of these observations suggested that the intracellular context in which IL-6 was activating ERK could be important.

Thus, we decided to investigate whether IL-6 was concomitantly activating additional pathways in MoDCs that could explain the differential reactivation phenotype between the two cell types. To do this, comparative phosphoproteomics of IL-6–stimulated MoLCs and MoDCs were performed (Fig. 1*e* and Fig. S1). This confirmed that MoLCs were responsive to IL-6, with evidence of both ERK and Stat activation detected (Fig. S1). Although the pattern of phosphorylation was broadly similar, a number of differences in protein phosphorylation profiles were observed between the two cell types in response to IL-6 (Fig. 1*f* and Fig. S1). We noted that the biggest changes in protein phosphorylation were observed with two SFKs, HCK and Src itself; thus, we investigated this family of kinases further.

### SFK activity is required for chromatin remodeling in response to IL-6

**Figure 2.**
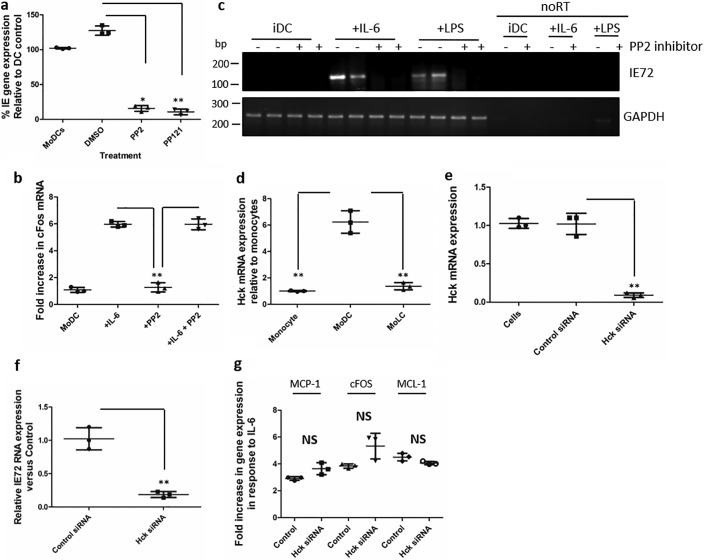
**SFK inhibitors and HCK depletion from cells reduces reactivation in experimental and natural latency.** *a*, experimentally latent MoDCs were incubated with medium (DCs), DMSO, or the SFK inhibitors PP2 and PP121 for 3 h and then stimulated with IL-6 and analyzed for IE RNA expression by qRT-PCR 16 h later. *b*, qRT-PCR for c-fos mRNA expression was performed in unstimulated immature DCs, or immature DCs pretreated with PP2 or a solvent control prior to IL-6 stimulation. *c*, naturally latent monocytes isolated from two seropositive donors were differentiated *in vitro* to MoDCs, pretreated with DMSO (−) or PP2 inhibitor (+) for 3 h, and stimulated with IL-6 or lipopolysaccharide (*LPS*) for 16 h, and complementary DNA or RNA (no reverse transcription control) was analyzed using a nested PCR for IE expression. *d*, HCK RNA expression was analyzed in monocytes, MoDCs, and MoLCs by qRT-PCR. *e–g*, MoDCs incubated with control or HCK-specific siRNAs were analyzed for HCK RNA expression 48 h after transfection (*e*) or stimulated with IL-6 and analyzed by qRT-PCR for IE (*f*) or cellular (*g*) gene expression 16 h later. *, *p* < 0.05; **, *p* < 0.01; *NS*, not significant.

To find out whether SFK activity was important for HCMV reactivation, we tested the effect of SFK inhibitors on HCMV reactivation from latency. Pretreatment of latently infected MoDCs with SFK inhibitors prior to IL-6 was sufficient to reduce IL-6–induced IE gene expression from both experimental and natural latency when analyzed 16 h after stimulation (Fig. 2, *a* and *c*). No comparable effect on IE gene expression was evident in primary infection of MoDCs or fibroblasts (Fig. S2), nor was IL-6–induced expression of c-fos mRNA affected in reactivating MoDCs (Fig. 2*b*), suggesting that pan-inhibition of IL-6–responsive genes was not occurring and, furthermore, that the requirement for SFK activity to drive viral IE gene expression was restricted to latency and reactivation.

Unlike Src, which is expressed in most cells, HCK expression is restricted to hematopoietic cells (22). Coupled with the observation that HCK expression was higher in myeloid cells that support HCMV reactivation (Fig. 2*d*), we chose to investigate HCK further. Using siRNAs, we transiently depleted HCK from MoDCs (Fig. 2*e*). Loss of HCK resulted in a clear decrease in IE gene expression in response to IL-6 (Fig. 2*f*). Again, the effect on IE RNA did not reflect global shutdown of IL-6–induced gene expression, as a number of other IL-6–responsive genes were relatively unaffected in HCK knockdown cells (Fig. 2*g*). Thus, HCK activity was an important component of SFK-induced reactivation of HCMV and was having a particularly strong effect on HCMV gene expression in MoDCs.

The observed inhibition of IE gene expression in our studies suggested that SFK/HCK activity was particularly important during the initial stages of reactivation, when a key viral promoter (the MIEP) must be activated. An important mechanism of regulation at the MIEP involves the activity of higher-order chromatin structure (6, 16, 23, 24). Most recently, we linked changes in chromatin structure required for reactivation with ERK-driven activation of cellular kinases, mitogen, and stress-activated kinases, which promote histone phosphorylation at the MIEP in a CREB transcription factor–dependent manner in MoDCs (16). Indeed, this concept of histone phosphorylation for reactivation of viral gene expression is an emerging theme in herpes virology (25, 26).

**Figure 3.**
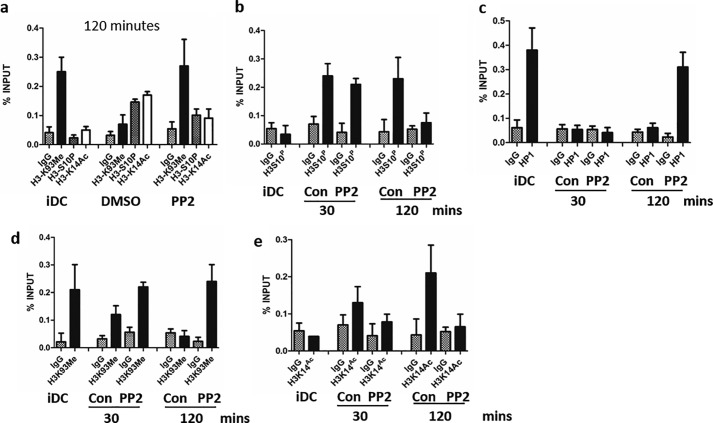
**SFK activity is required for formation of transcriptionally active chromatin at the MIEP.** *a*, ChIP assays were performed with antibodies against histone H3 trimethylated on lysine 9 (*H3K93me*), phosphorylated on serine 10 (*H3S10P*), or acetylated on lysine 14 (*H3K14Ac*) or with an isotype control on unstimulated MoDCs (*iDC*) or 2 h after IL-6 stimulation after prior treatment with DMSO or PP2 for 1 h. DNA was amplified in an MIEP qPCR, and signal was expressed relative to input (*n* = 3). *b–e*, ChIP assays were performed 30 and 120 min after IL-6 stimulation with antibodies against heterochromatin protein 1b (*HP1*), histone H3 trimethylated on lysine 9, phosphorylated on serine 10, or acetylated on lysine 14 or with an isotype control (*Con*) 30 min or 2 h after IL-6 stimulation after prior treatment with DMSO or PP2 for 1 h. DNA was amplified in an MIEP qPCR, and signal was expressed relative to input (*n* = 2).

Given the importance of chromatin in viral reactivation, we asked what, if any, effect SFK inhibitors had on the chromatin phenotype at the MIEP (Fig. 3). As expected, in reactivating MoDCs stimulated with IL-6 histone, H3S10 phosphorylation and H3K14 acetylation predominated at the reactivating MIEP and was concomitant with a reduction in H3K9 methylation (Fig. 3*a*). In contrast, 2 h after IL-6, in the presence of the SFK inhibitor PP2, the chromatin phenotype copied the latent MIEP phenotype prior to IL-6 addition (Fig. 3*a*). This suggested that SFK activity could be co-involved in H3S10 phosphorylation with ERK.

However, analysis of the MIEP at earlier time points revealed that, 30 min after IL-6 H3S10, phosphorylation at the MIEP was evident even in PP2-treated cells despite being reversed by 2 h (Fig. 3*b*). This transient H3S10 phosphorylation was concomitant with the loss of HP1 from the MIEP (Fig. 3*c*); H3S10 phosphorylation has been hypothesized to promote HP1 dissociation from methylated histones (27). Indeed, few differences in chromatin phenotype at the MIEP were observed between control and PP2-treated MoDCs at 30 min, except histone H3K14 acetylation was reduced (Fig. 3, *d* and *e*). This suggested a model whereby ERK was initiating chromatin modifications at the MIEP, but additional cellular signaling (*i.e.* SFK signaling) was required to potentiate the effects after ERK.

### The MOZ histone acetyltransferase promotes HCMV reactivation in a SFK-dependent manner

**Figure 4.**
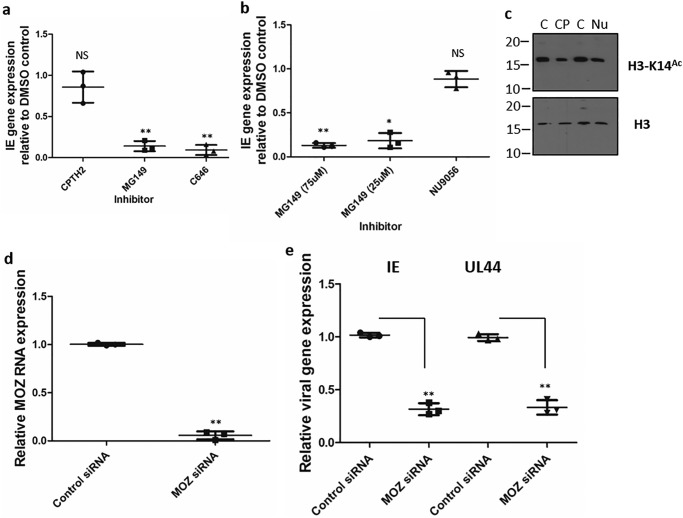
**HCMV reactivation depends on MOZ histone acetyltransferase activity.** *a* and *b*, experimentally latent MoDCs were incubated with medium (DCs), DMSO, or inhibitors of p300, Tip60/MOZ (MG149, 75 μm), or MOZ (MG149, 25 μm) for 3 h and then stimulated with IL-6 and analyzed for IE (*a*) and UL44 (*b*) RNA expression by qRT-PCR 16 h later. *c*, Western blot for MoDCs treated with histone H3 (*H3*) or histone H3 acetylation on lysine 14 (*H3K14Ac*^)^ in DMSO (*C*), CPTH2 (*CP*), and NU9056 (*Nu*). *d* and *e*, MoDCs incubated with control or MOZ-specific siRNAs were analyzed for MOZ RNA expression 48 h after transfection (*d*) or subsequently stimulated with IL-6 and analyzed by qRT-PCR for IE (*e*) and UL144 gene expression 16 h later. *, *p* < 0.05; **, *p* < 0.01; *NS*, not significant.

To test the hypothesis that ERK is important for initiating reversal of the chromatin phenotype but that the effect is potentiated by SFK activity, we sought to identify the histone acetyltransferase responsible for HCMV reactivation because this would likely be involved at the MIEP after ERK-mediated histone phosphorylation. Although the focus of our study is on HCMV reactivation in MoDCs, we chose to include an analysis of reactivation in macrophage-like cells to test whether the same HAT was responsible in multiple cell types that support HCMV reactivation. To do this, we used pharmacological inhibitors of cellular HAT activity in two models of HCMV reactivation: our MoDC model and also the THP1 macrophage model. In the THP1 model cell line, we observed that MG149 (an inhibitor of MOZ and Tip60 HATs) reduced IE gene expression (Fig. S3*a*). A titration of the MYST family inhibitor MG149 (which has different activities depending on concentration) and the relative inability of a Tip60-specific inhibitor (NU9056) to inhibit IE gene expression suggested that MOZ was the HAT most important for reactivation in THP1 cells (Fig. S3*b*). Interestingly, in the MoDC model, the phenotype was different. In these cells, inhibitors of p300 (C646) and MYST family HATs (MG149) had the biggest effect on IE gene expression (Fig. 4*a*). Again, titration of MG149 alongside a lack of activity of NU9056 suggested that it was MOZ, and not Tip60, that was important for reactivation in MoDCs (Fig. 4*b*). Furthermore, the failure of CPTH2 and NU9056 to prevent reactivation was not due to these inhibitors reducing histone acetylation in cells (Fig. 4*c*). However, the data obtained using C646 also suggested that p300 activity appeared to be more important for reactivation in the MoDC model, in contrast with the THP1 macrophage cell line, where very little effect was observed with C646 (Fig. S3). This MoDC-specific effect on reactivation with C646 could be recapitulated with an inhibitor of the transcription factor CREB (66615), a known interaction partner of p300 (Fig. S4). Specifically, 66615 inhibited reactivation in MoDCs but not THP1 cells (Fig. S4*a*), despite 66615 being active in the THP1 cell line (Fig. S4*b*).

For the purpose of this study, we chose to focus on the suggestion that MOZ activity is centrally important in both models of HCMV latency and reactivation tested. However, although the pharmacological data argued for a role of MOZ, we sought to confirm this genetically prior to further investigation of the effect of MOZ on HCMV reactivation. To do this, MoDCs were transfected with MOZ-targeted siRNAs or a nontargeting control, and 48 h later, reactivation was stimulated with IL-6. The data demonstrate that depletion of MOZ from DCs (Fig. 4*d*) was sufficient to reduce viral IE gene expression in response to IL-6 (Fig. 4*e*); thus, pharmacological and genetic depletion of MOZ activity in MoDCs was sufficient to prevent HCMV reactivation.

**Figure 5.**
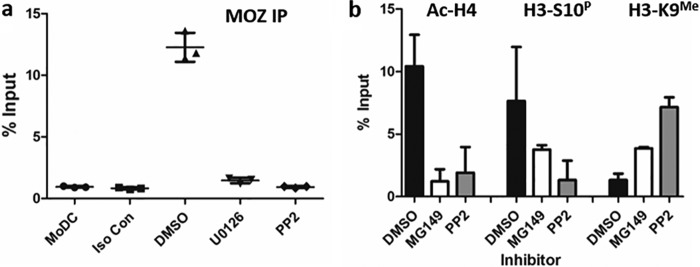
**MOZ recruitment to the MIEP depends on SFK signaling and is required for histone H4 acetylation.** *a*, ChIP assays were performed on MoDCs using antibodies against MOZ or an isotype control 1 h after IL-6 stimulation after prior treatment with DMSO, ERK (U0126), or SFK (PP2) inhibitors for 1 h. DNA was amplified in an MIEP qPCR, and signal was expressed relative to input (*n* = 3). *b*, ChIP assays were performed with antibodies against histone H3 trimethylated on lysine 9 (*H3K9me*) and phosphorylated on serine 10 (*H3S10P*) and pan-acetylated histone H4 on MoDCs or 2 h after IL-6 stimulation after prior treatment with DMSO, SFK (PP2), or MOZ (MG149) inhibitors for 1 h. DNA was amplified in an MIEP qPCR, and signal was expressed relative to input (*n* = 2).

The demonstration that MOZ was important for reactivation did not determine whether the effects were direct or indirect. To begin to address this, we utilized ChIP analyses to demonstrate that MOZ was bound to the MIEP during the early stages of viral reactivation, arguing that it played a direct role in the regulation of IE gene expression (Fig. 5*a*). Furthermore, pretreatment with the SFK inhibitor PP2 prevented recruitment of MOZ to the MIEP, suggesting that activation of SFK signaling was driving the recruitment of MOZ (Fig. 5*a*). Consistent with MOZ binding being important for histone modifications occurring at the MIEP during reactivation, it was demonstrated that acetylation of histone H4 at the MIEP was markedly reduced in MG149-treated cells 2 h after IL-6 (Fig. 5*b*). Furthermore, it was clear from the analyses that histone demethylation or prolonged histone phosphorylation were only partially impaired, suggesting that these events preceded or were independent of MOZ activity (Fig. 5*b*).

## Discussion

These data reinforce the view that HCMV reactivation is highly dependent on cellular mechanisms of gene regulation. Furthermore, they demonstrate a novel role for SFK signaling in the control of gene expression via chromatin modifications. Specifically, we show that ERK–MAPK–driven HCMV reactivation depends on the concomitant activation of SFK signaling in response to IL-6. A key event for reactivation is the availability and activation of HCK, a hematopoietic cell–specific kinase that is up-regulated upon myeloid DC differentiation. In light of our current data, we propose a model whereby SFK activity works after ERK-mediated histone phosphorylation to support transition of the MIEP to a fully open chromatin state to ensure robust IE gene expression and, ultimately, viral reactivation. Furthermore, we propose that this dependence on SFKs is linked with a role in the recruitment of MOZ HAT activity to the MIEP to drive chromatin modifications conducive for transcription.

The data emphasize how cell signaling and the regulation of eukaryotic gene expression are highly context-specific. ERK–MAPK activity can drive HCMV reactivation (8, 16), but what we now show is that it is the context in which the ERK pathway is activated that is important. Specifically, ERK is only pro-reactivation when additional signaling, and specifically HCK, is activated at the same time by IL-6. What became evident was that this concomitant signaling pathway activation by IL-6 was most effective in MoDCs, providing a model for the differential regulation of gene expression in a cell type–specific manner.

An important role of cell identity also resonated with attempts to identify the HAT responsible for HCMV reactivation, which again had elements of cell-specific responses. For instance, a role of p300 would be consistent with the known interaction of this protein with phosphorylated CREB (28), a factor we have reported to be required for efficient reactivation of HCMV in response to IL-6 in MoDCs (16). Indeed, it is noteworthy that the C646 inhibitor phenotype was recapitulated with the CREB transcription factor inhibitor (66615), suggesting that an interaction between these two proteins was driving the MoDC reactivation phenotype. In contrast, the C646 data suggest that p300 activity is less important in the THP1 macrophage model, where, instead, MOZ activity appeared to be sufficient. A likely explanation is that activation of the MIEP in the THP1 model is via the activation of different pathways, which is dictated by the stimuli used for reactivation. For instance, multiple members of the MYST family of HATs, including Tip60 and MOZ, interact with the NF-κB pathway (29, 30). The phorbol ester used to promote THP1 differentiation is a potent activator of NF-κB–dependent transcription (31), which may be sufficient in macrophage-like cells to reactivate HCMV. Thus, the virus has evolved to utilize different transcription and signaling pathways to facilitate reactivation in multiple myeloid cell types in response to a number of different ligands.

However, the role of MOZ as a player in both cell types suggests that events common to both are required. MOZ histone acetyltransferase activity is heavily implicated in the hematopoietic stem cell differentiation processes, to which HCMV reactivation is tied (32). Pertinent for our studies, MOZ has been shown to target lysine residues 9 and 14 on H3 for acetylation (32) and also histone H4, which is heavily acetylated when bound to the MIEP in reactivating cells (6). Lysine 9 methylation is a major driver of HP1 recruitment, and its binding is also correlated with regulation of the MIEP (6, 33). Furthermore, H3K9 modifications are closely tied with the nearby acetylation of H3K14 residues. Indeed, here we observed that inhibition of SFK activity resulted in failure to reverse H3K9 methylation and promote H3K14 acetylation, potentially providing a link between SFKs, MOZ, and HCMV reactivation. Furthermore, the requirement for MOZ activity in both models may reflect a combinatorial role in HAT activity. Indeed, activation of AML1 transcriptional activity has been shown to involve MOZ and p300 acting co-operatively to enhance AML1 function; thus, a similar model may be at play here (34).

Another aspect of the analogy to AML1 that is also interesting is that MOZ activity has been linked to the development of acute myeloid leukemia (AML); the MOZ gene translocates to generate gene fusions with other transcriptional regulators (including p300) (34, 35). Some studies show that AML patients with HCMV have better clinical outcomes. Although the mechanistic basis for this is not understood, one hypothesis is that the tumor cells express CMV antigens that promote immune clearance. Potentially, the over-activation of MOZ in that could occur in some AML cells (depending on the genetic basis of the gene fusion (35)), and elevated activity on the MIEP is driving this surprising benefit of HCMV infection through production of more IE antigen targets for T cells or indirectly by promoting recruitment of cytotoxic T cells to the tumor environment.

This study has focused on cellular mechanisms of regulation, not addressing the contribution of latent viral functions. However, US28, another latently expressed gene product, has the capacity to have a profound effect on cell signaling pathways in a cell type–specific manner (36). Many of these pathways have already been implicated in regulation of the MIEP, including ERK, mitogen and stress activated kinase, NF-κB, and, most recently, cFos (37, 38). Notably, two other prominent hits in one study were Src and HCK, with US28 enhancing their phosphorylation during latent infection (37). If activatory, then how this is reconciled with a role for HCK in reactivation is not clear. It is possible that the phosphorylation was inhibitory; thus, it could be a case of US28 turning them off. Alternatively, it could suggest that the pathway by which HCK is activated is crucial. HCK can be activated via TLR4 or CD45 signaling at the plasma membrane or via gp130 activation by IL-6. and, pertinently, the mechanism of Hck activation can result in quite different outputs downstream of Hck in the same cells (22). US28 is a membrane-bound protein (36). Thus, it could be hypothesized that activation via US28 is gp130-independent and, furthermore, that the virus is commandeering Hck activity during latency for other functions. This could potentially deprive the cell of available Hck that can be activated by gp130 signaling in response to IL-6 in latent cells. However, given that MoDC differentiation promotes an increase in Hck expression, it may suggest that increased availability of *de novo* Hck makes MoDCs more responsive to IL-6 in terms of HCMV reactivation.

Taken together, these data further demonstrate, through a study of viral gene regulation, how integrated signaling through multiple pathways promotes cell type– and ligand-specific gene expression and imply a clear role of SFK activity in the recruitment and activity of chromatin-modifying enzymes to promoters to differentially control gene expression. They also exemplify the benefit of studying how persistent viruses are dependent on these host pathways to complete their lifecycle, as it provides a highly tractable tool to study the mechanisms of eukaryotic gene regulation. Importantly, they further demonstrate the dependence on host cell functions of pathogens, which, in this case, may expose the virus to novel host-targeted strategies to control the reactivation of this highly dangerous pathogen in immune-suppressed patients.

## Experimental procedures

### Ethics statement

Collection of venous blood samples from anonymous donors was approved and performed in accordance with established guidelines for the handling and processing of said tissue by the University College London Local Research Ethics committee. All studies with human material abided by Declaration of Helsinki principles.

### Virus, cell lines, culture, and reagents

The clinical isolate Merlin (39) was purified from infected human retinal pigment epithelial cells using sorbitol gradients as described previously (40). Viruses for these studies were characterized by their ability to infect primary dendritic cells to assay myelotropism. Routinely, virus preparations infected 10%–20% DCs when used at an m.o.i. of 5, calculated on fibroblasts.

Primary CD14+ monocytes were isolated from venous blood of anonymous donors who had given informed consent under the appropriate local rules. Typically, 50 ml of venous blood was diluted in PBS 1:1 and then separated on a Ficoll gradient (Lymphoprep, Nycomed, Melville, NY). Labeled CD14+ cells (Miltenyi Biotec, Auburn, CA) were rescued using magnet-activated cell sorting and cultured in X-vivo 15 (Cambrex, Walkersville, MD) supplemented with 2 mm l-glutamine and cytokines to promote DC differentiation where appropriate. Recombinant adenoviruses expressing constitutively active MEK1 and GFP were purchased and used directly without propagation at an m.o.i. of 100 (Cell Biolabs, San Diego, CA).

### Experimental infection of primary cells and differentiation

CD14+ monocytes (5 × 10^5^ cells/well) were infected in 250 μl of X-vivo 15 medium plus HCMV (m.o.i. = 5) for 3 h and then rescued in 1 ml of fresh medium. After 3 days, experimentally infected CD14+ cells were differentiated to immature MoDCs or immature MoLCs as described previously (8, 21). Briefly, CD14+ monocytes were cultured with interleukin-4 (100 ng/ml) and GM-CSF (100 ng/ml) for 6 days to promote differentiation to a MoDC phenotype or IL-4 (10 ng/ml), GM-CSF (100 ng/ml), and transforming growth factor β (10 ng/ml) for 2 days, followed by a further 4 days with GM-CSF and transforming growth factor β to promote an MoLC phenotype. Following culture, immature MoLCs and MoDCs were incubated with recombinant IL-6 (500 ng/ml). All cytokines were from Peprotech (Rocky Hill, NJ) unless otherwise stated.

THP1 cells were cultured in RPMI 1640 medium supplemented with 10% fetal bovine serum. To establish latent infection, monocytes or THP1 cells were infected for 3 h with Merlin at an m.o.i. of 5 (based on human foreskin fibroblasts infectivity). Monocytes were left for 3 days before differentiation into MoDCs. THP1 cells were left for 5 days before addition of phorbol 12-myristate 13-acetate (20 ng/ml, Sigma) for 24 h to trigger THP1 differentiation and HCMV reactivation.

### Inhibitors, antibodies, and chromatin immunoprecipitation

To test for inhibition of HCMV reactivation, inhibitors were added prior to IL-6 stimulation: MEK/ERK, U0126 or the inactive analog U0124) (Calbiochem, both 10 μm); SFK, PP2 (Sigma) or PP121 (Santa Cruz Biotechnology) (both 20 nm); p300, C646 (Sigma, 25 μm); MYST family, MG149 (Axon Medchem; 75 μm); Gcn5, CPTH2 (Sigma, 100 μm); Tip60, NU9056 (Tocris, 10 μm); and MOZ, MG149 at 25 μm as directed by the manufacturer. CREB (666-15, Calbiochem) was used at 1 μm in DMSO.

For Western blotting, rabbit anti-p42/p44 or rabbit anti-phospho-p42/p44 antibodies (ERK) or rabbit anti-GAPDH (1:1,000, Abcam) were used, followed by HRP-conjugated goat anti-rabbit antibody (1:4,000, Santa Cruz Biotechnology, Santa Cruz, CA). To analyze histone modifications by Western blotting, an anti-acetylated lysine 14 H3 rabbit antiserum (Millipore, 1:1,000 dilution) or rabbit anti-histone H3 antibody (Millipore, 1:1,000 dilution) was used and detected as for ERK above.

For ChIP studies, DCs were fixed with 1% formaldehyde (10 min) and then prepared for ChIP using an enzymatic digestion kit as described by the manufacturer (Chromatin Prep Module, Thermo Fisher Scientific). DNA fragments associated with histones were immunoprecipitated with rabbit IgG (Sigma), anti-phospho-histone H3S10 antiserum (ChIP-grade, 1:200 dilution), anti-trimethyl lysine 9 histone H3 antiserum (ChIP-grade, 1:200), anti-acetylated lysine 14 H3 antiserum (Millipore, 1:200 dilution), anti-acetylated histone H4 (ChIP-grade, 1:200 dilution), anti-HP1b (Abcam, 1:100 dilution) and anti-MOZ (Abcam, 1:100 dilution); all antibodies were from Upstate Biotechnology (Charlottesville, VA) unless stated otherwise. For detection of the MIEP of HCMV, DNA from disrupted nucleosomes was precipitated and amplified by qPCR using 5′-CCA AGT CTC CAC CCC ATT GAC (sense) and 5′-GAC ATT TTG GAA AGT CCC GTT G (antisense) primers and quantified with SYBR Green. Specific immunoprecipitation of sequences was expressed as enrichment from input control.

For FACS analyses, 10^5^ cells were pelleted at 400 × *g* for 5 min and then resuspended in the residual volume. The cells were incubated with 3 μl of phycoerythrin-conjugated mouse anti-human IL-6 receptor antibody or a fluorochrome-conjugated mouse IgG1 isotype control for 20 min in the dark. Following washing in 10× volumes of PBS, the cells were pelleted at 400 × *g* for 5 min and resuspended in 500 μl of PBS before analysis by flow cytometry (BD FACSCalibur or BD FACSSort). Data handling was performed using WinMDI2.9 software. All antibodies were from BD Life Sciences (Franklin Lakes, NJ) unless stated otherwise.

### Nucleic acid isolation, reverse transcription, and qPCR

RNA was isolated from 10^6^ cells using RNAeasy spin columns as described by the manufacturer (Qiagen, Valencia, CA). Following isolation, total RNA was incubated with DNase I (Promega, Madison, WI) and then amplified by qRT-PCR. Gene expression was quantified by SYBR Green (Qiagen) using the following primer pairs: IE, 5′-CAC GAC GTT CCT GCA GAC TAT G and 5′-CAT CCA CAT CTC CCG CTT AT; UL44, 5′-GTA CAA CAG CGT GTC GTG CT and 5′-ATA ACC GCG TCA GTT TCC AC; cFOS, 5′-GAA TAA GAT GGC TGC AGC CAA ATG GCC CGC AA and 5′-CAG TCA GAT CAA GGG AAG CCA CAG ACA TCT; MCP-1, 5′-TGG AAT CCT GAA CCC ACT TC and 5′-CCC AGT CAC CTG CTG TTA T; MCL-1, 5′-TGC AGG TGT GTG CTG GAG TAG and 5′-GCT CTT GGC CAC TTG CTT TTC; HCK, 5′-CAT CAT CGT GGT TGC CCT GTA and 5′-GCG GGC GAC ATA GTT GCT T; 18S, 5′-GTA ACC CGT TGA ACC CCA and 5′-CCA TCC AAT CGG TAG TAG CG. MOZ sequences were amplified using MOZ primer set B (Santa Cruz Biotechnology). Samples were amplified using an ABI 7500 Fast Real Time PCR machine (Applied Biosystems, Foster City, CA; 95 °C for 15 s and 60 °C for 45 s).

To amplify viral DNA from naturally infected cells, nested IE primers were used in a standard PCR with the following cycling conditions: 95 °C (5 min); then 20 cycles of 94 °C (1 min), 55 °C (40s), and 72 °C (40s); and then a final extension at 72 °C for 10 min. Then 5 μl of this product was amplified under the same conditions for 20 cycles using nested primers. IE72 was amplified with the sense primer 5′-CAT CCA CAT CTC CCG CTT AT-3′ and the antisense primer 5′-CAC GAC GTT CCT GCA GAC TAT G-3′, followed by nested PCR with the sense primer 5′-GCG CCA GTG AAT TTC TCT TC and the antisense primer 5′-ACG AGA ACC CCG AGA AAG ATG, yielding a final nested product of 302 bp (DNA) or 131 bp (complementary DNA).

### Proteomics arrays

A phosphoproteomics array that measured phosphorylation of 43 human kinases involved in cell signaling was used as described by the manufacturer (R&D Systems, Minneapolis, MN). Briefly, lysates from unstimulated and IL-6–stimulated MoDCs or MoLCs were incubated with the arrays for 3 h and developed using ECL and autoradiography. Pixel density was measured using ImageJ software (National Institutes of Health), and the -fold change was expressed from untreated control cells *versus* IL-6–treated cells.

### siRNA depletion of gene expression in DCs

MoDCs were cultured in X-vivo 15 medium (serum-free) and then transfected with HCK or MOZ siRNAs using Lipofectamine 2000 (Invitrogen) as described by manufacturer. Pooled HCK siRNAs (Hck 4392420, Ambion) or MOZ siRNAs (sc-37959, Santa Cruz Biotechnology) were transfected into DCs and then knockdown was confirmed 48 h after transfection using gene-specific primers. Controls were nontargeting primers obtained from the same supplier as the targeting siRNAs (Ambion and Santa Cruz Biotechnology).

### Statistical analyses

A Mann–Whitney *U* test was applied to test for significance between the means, as a nonparametric distribution was assumed. Statistical analyses were only applied when *n* > 2. All scatterplots depict the mean and 1 S.D. Significance was assumed when *p* < 0.05.

## Author contributions

L. Dupont and M. B. R. conceptualization; L. Dupont, L. Du, M. P., S. C., M. M., and M. B. R. data curation; L. Dupont and M. B. R. formal analysis; L. Dupont, L. Du, M. P., S. C., M. M., and M. B. R. investigation; M. B. R. supervision; M. B. R. funding acquisition; M. B. R. validation; M. B. R. methodology; M. B. R. writing-original draft; M. B. R. project administration; M. B. R. writing-review and editing.

## Supplementary Material

Supporting Information

## Footnotes

3 The abbreviations used are:

HCMV: human cytomegalovirus
MIEP: major immediate-early promoter
IE: immediate-early
ERK: extracellular signal-regulated kinase
MAPK: mitogen-activated protein kinase
SFK: Src family kinase
MoDC: monocyte-derived dendritic cell
MoLC: monocyte-derived Langerhans cell
HCK: hematopoietic cell kinase
MOZ: monocytic leukemia zinc finger protein
CREB: cAMP response element–binding protein
HAT: histone acetyltransferase
AML: acute myeloid leukemia
m.o.i.: multiplicity of infection
H3S10: histone H3 serine 10
H3K9: histone H3 lysine 9
H3K14: histone H3 lysine 14
MYST: MOZ, Ybf2, Sas2, Tip60 family
RT-qPCR: quantitative RT-PCR.
