# Supplementary material for: Src family kinase activity drives cytomegalovirus reactivation by recruiting MOZ histone acetyltransferase activity to the viral promoter
Source: J Biol Chem. 2019 Jul 4;294(35):12901–10. doi: 10.1074/jbc.RA119.009667 (PMC6721939; doi:10.1074/jbc.RA119.009667)
Supplement: Supporting Information [file supp_294_35_12901__index.html]

Src family kinase activity drives cytomegalovirus reactivation by recruiting MOZ histone acetyltransferase activity to the viral promoter — SFK activity promotes MOZ recruitment to a viral promoter — Src family kinase activity drives cytomegalovirus reactivation by recruiting MOZ histone acetyltransferase activity to the viral promoter — SFK activity promotes MOZ recruitment to a viral promoter — Supporting Information 

# Src family kinase activity drives cytomegalovirus reactivation by recruiting MOZ histone acetyltransferase activity to the viral promoter

## Supporting Information

- Supporting Information (to be published online) - Supporting data
