# Supplementary material for: Src family kinase activity drives cytomegalovirus reactivation by recruiting MOZ histone acetyltransferase activity to the viral promoter
Source: J Biol Chem. 2019 Jul 4;294(35):12901–10. doi: 10.1074/jbc.RA119.009667 (PMC6721939; doi:10.1074/jbc.RA119.009667)
Supplement: Supporting Information [file supp_RA119.009667_153336_1_supp_355343_ptth9k.pdf]

Src family kinase actvity drives cytomegalovirus reactivation through recruitment of MOZ histone acetyltransferase activity

Liane Dupont[#], Lily Du, Madeleine Poulter, Stephanie Choi, Megan McIntosh & Matthew B. Reeves*

Supplementary figures

Figure S1 Summary of phosphoproteomic analysis for IL-6 induced changes in phosphorylation

Figure S2 Pre-treatment of permissive cells with SFK inhibitors has no impact on infection

Figure S3 MG149 inhibits the histone acetyltransferase activity required for HCMV reactivation in THP1 macrophage-like cells

Figure S4 A CREB inhibitor blocks reactivation in MoDCs but not the THP1 cell line

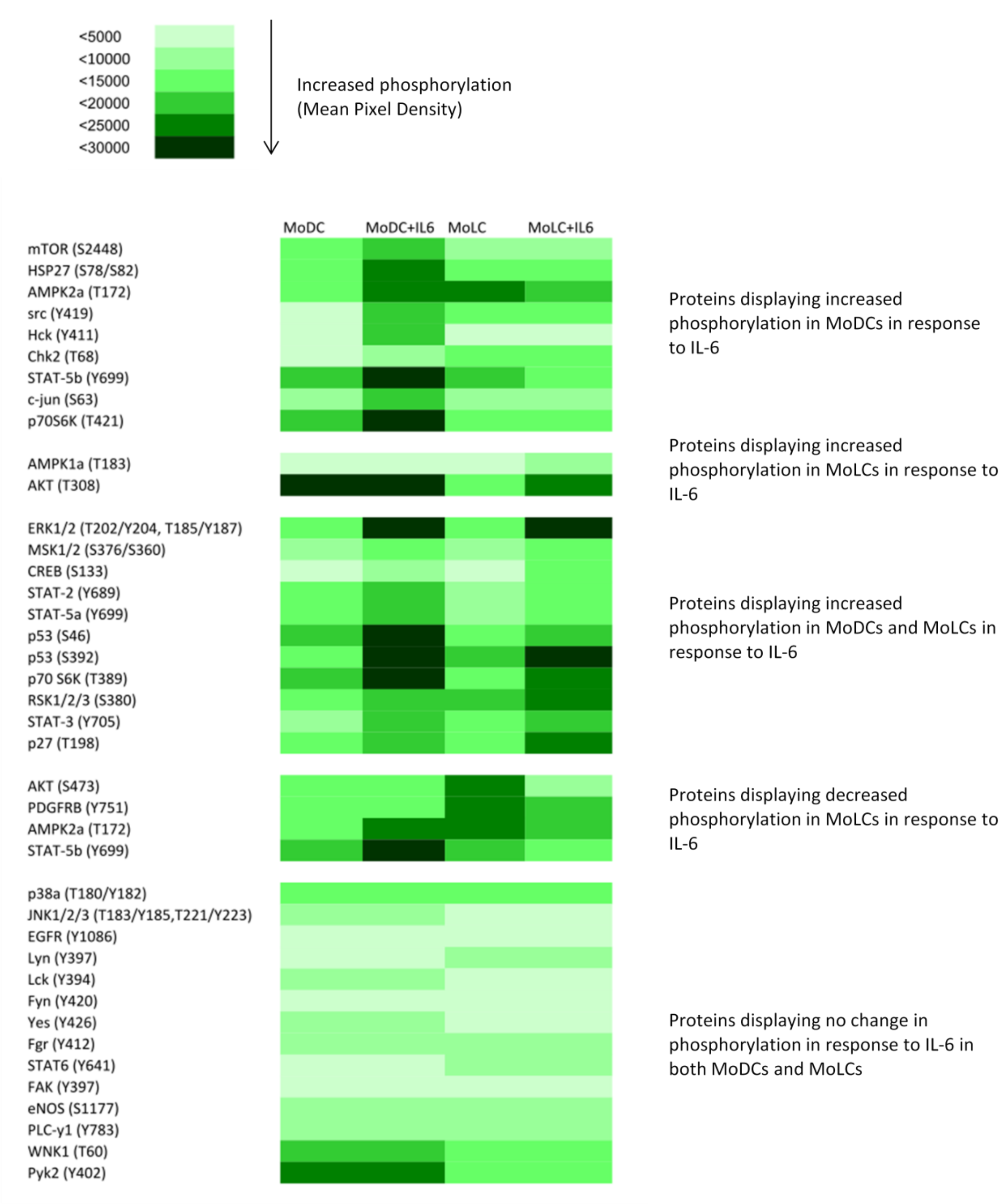

Figure S1 Summary of phosphoproteomic analysis for IL-6 induced changes in phosphorylation A heatmap analysis of changes to protein phosphorylation in MoDCs and MoLCs upon stimulation with IL-6. Proteins were grouped according to common changes in protein phosphorylation in both cell types and into cell type specific changes that occurred.

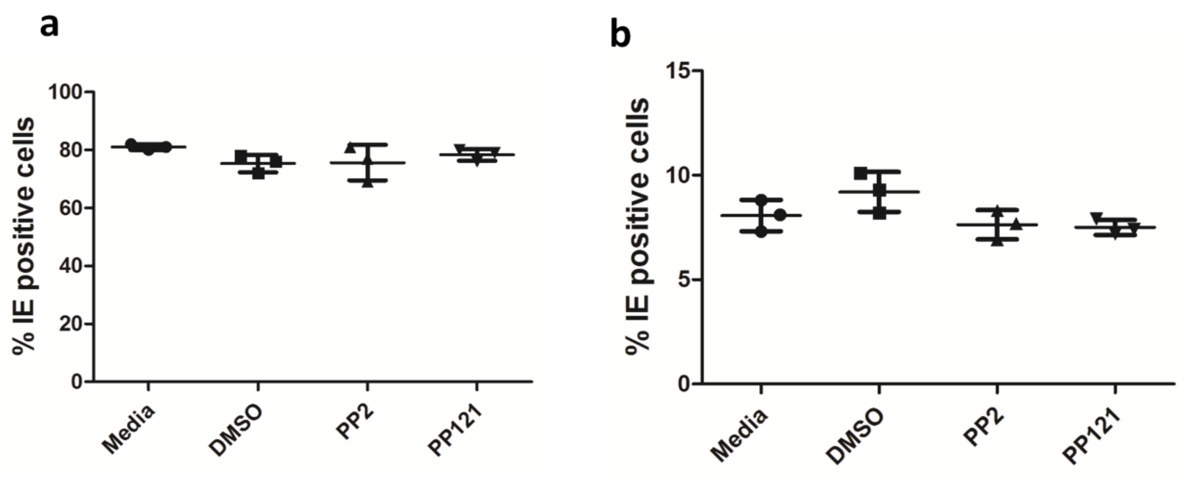

Figure S2 Pre-treatment of permissive cells with SFK inhibitors has no impact on infection A,B) Human fibroblasts (A) or MoDCs (B) were pre-incubated with media (-) DMSO, or SFK inhibitors PP2 and PP121 for 1 hour then infected with HCMV. At 8hpi, cells were fixed and stained for IE gene expression and scored for % infection using Hermes WiScan automated counting. (n=3)

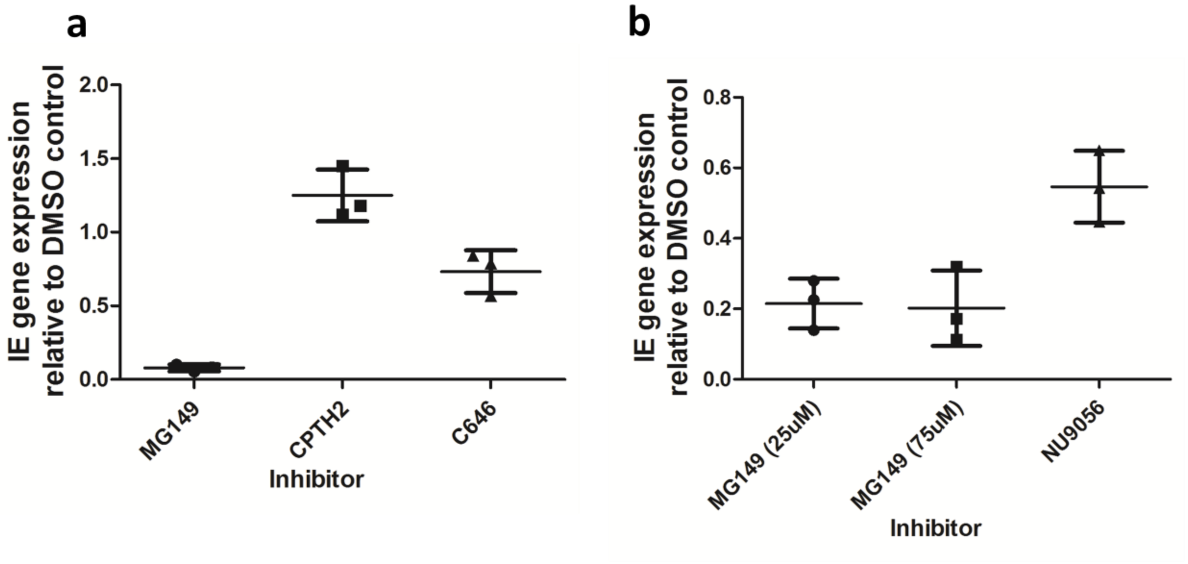

Figure S3 MG149 inhibits the histone acetyltransferase activity required for HCMV reactivation in THP1 macrophage-like cells A) Experimentally latent THP1 cells were incubated with DMSO or histone acetyltransferase inhibitors MG149 (Tip60/MOZ), CPTH2 (Gcn5) and C646 (p300) inhibitors for 3 hours then stimulated with PMA and analysed for IE or 18S RNA expression by qRT-PCR 16 hours later. **B)** Experimentally latent THP1 cells were incubated with DMSO or histone acetyltransferase inhibitor MG149 at 75uM (Tip60/MOZ) and 25uM (MOZ), or NU900566 (Tip60) inhibitors for 3 hours then stimulated with PMA and analysed for IE or 18S RNA expression by qRT-PCR 16 hours later. Change in IE gene expression was quantified using the $2^{-\Delta\Delta CT}$ method (n=3).

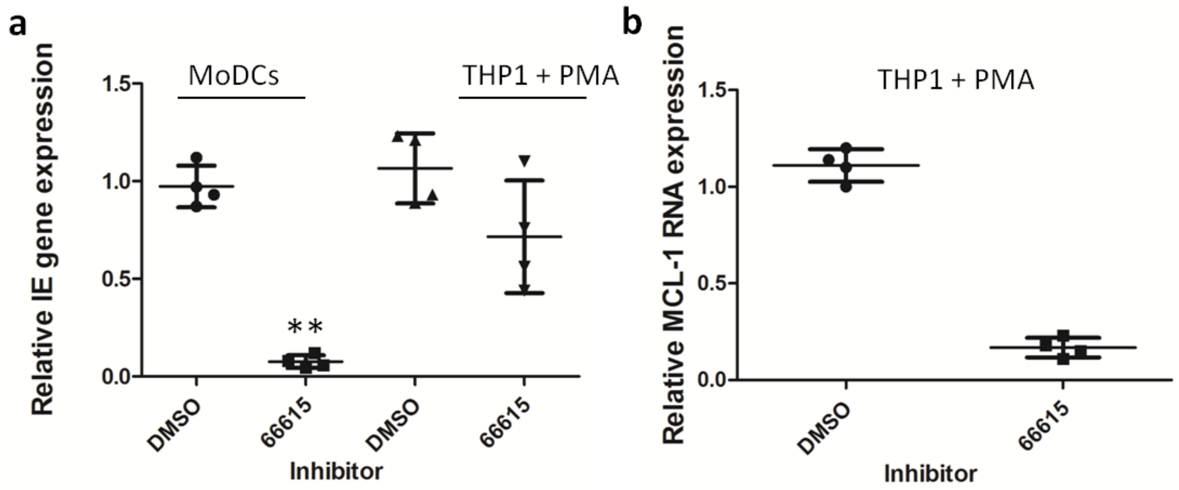

Figure S4 A CREB inhibitor blocks reactivation in MoDCs but not the THP1 cell line

A) MoDCs or THP1 cells were pre-treated with a CREB inhibitor (66615) or DMSO control prior to the addition of IL-6 (in MoDCs) or PMA (THP1 cells) to promote reactivation. IE gene expression was measured by qRT-PCR 24 hours post stimulation and expressed relative to the untreated control cells. n=4 **$p<0.01$ **B)** THP1 RNA samples analysed in (A) were concomitantly analysed for MCL-1 gene expression by qRT-PCR 24 hours post stimulation and expressed relative to the untreated control cells. n=4 **$p<0.01$.
